# Genome-wide identification and functional characterization of wheat Brassinazole-resistant transcription factors in response to abiotic stresses and stripe rust infection

**DOI:** 10.3389/fpls.2023.1144379

**Published:** 2023-06-13

**Authors:** Peng Zhang, Hanwen Yan, Yu Liu, Yi Chai

**Affiliations:** Key Laboratory of Sustainable Crop Production in the Middle Reaches of the Yangtze River (Co-construction by Ministry and Province)/College of Agriculture, Yangtze University, Jingzhou, China

**Keywords:** *TaBZRs*, bioinformatics, phylogenetic analysis, group specificity, stripe rust

## Abstract

Brassinazole-resistant (BZR) transcription factors (TFs) are key players in brassinolides (BRs) signaling pathway, which is widely involved in regulating plant growth and development, as well as in plant responding to a variety stresses. Despite their critical roles, little is known about BZR TFs in wheat. In this study, we performed genome-wide analysis of BZR gene family from wheat genome, and 20 *TaBZRs* were identified. Based on the phylogenetic relationships of *TaBZR* and *BZRs* from rice and *Arabidopsis*, all *BZR* genes were clustered into four groups. The intron-exon structural patterns and conserved protein motifs of *TaBZRs* showed high group specificity. *TaBZR5*, 7, and 9 were significantly induced after salt, drought treatment, and stripe rust infection. However, *TaBZR16*, which was significantly upregulated under NaCl application, was not expressed during wheat-stripe rust fungus interaction. These results indicated that BZR genes in wheat play different roles in response to various stresses. The results of this study will lay a foundation for further in-depth functional studies of *TaBZRs* and will provide information for the breeding and genetic improvement of wheat against drought and salt stresses.

## Introduction

The brassinosteroids (BRs) are plant specific and are widely distributed among plants (Clouse and Sasse, 1998; Schaller, 2003; Agarwal et al., 2006). BRs play fundamental roles including regulating seed germination, cell growth, reproduction, senescence, and root growth (Nolan et al., 2020; Hu et al., 2021). Current studies have shown that BR mediates abiotic stresses in plants and play crucial role in resistance to temperature changes, salinity, drought, and organic pollutants (Vardhini and Anjum, 2015; Lee et al., 2018; Ahammed et al., 2020).

Brassinazole-resistant (BZR) transcription factors play important roles in the BR signaling pathway through regulating the expression of BR genes (Yin et al., 2005; Li et al., 2018; Yu et al., 2019). They positive regulate BR signaling and participate in a variety of stress response pathways (He et al., 2005; Miyaji et al., 2014; Singh et al., 2014; Li et al., 2017). In *Arabidopsis thaliana*, BZR1 and BRI1-EMS-SUPPRESSOR 1 (BES1) are well characterized members in the BZR family, with at least four other homologs designated as BES1/BZR1 Homolog1 (BEH1) to BEH4 (Yin et al., 2005; Sun et al., 2010). The N-terminal domain with DNA-binding activity of BZR protein is highly conserved (He et al., 2005; Yin et al., 2005). BZR proteins also contain glycosylation sites of the Glycogen synthase kinase 3 (GSK3), which consists of 22-24 amino acids. Besides, some BZR members also contain PEST (Proline-Glutamic acid-Serine-Threonine) motif to control protein stability (Yin et al., 2005).

BZR1 is a positive regulator of the BR signaling pathway and cooperates with light signal transcription factors to regulate cell elongation and plant growth (Li et al., 2001; Sun et al., 2010). Moreover, BZR1/BES1 positively regulated freezing tolerance by binding to the promoter of genes involved in both CBF-dependent and CBF-independent pathways in Arabidopsis (Li et al., 2017). It was reported that AtBES1 played a negative role in drought responses (Chen and Yin, 2017). On the contrary, TaBZR2 positively regulates drought responses by activation of TaGST1 (Xiao-Yu et al., 2019). Furthermore, TaBZR2 also conferred resistance to wheat stripe rust by activation of chitinase *Cht20.2* transcription (Bai et al., 2021).

Wheat (*Triticum aestivum* L.) is one of the most important food crops, and plays irreplaceable role in feeding the population of the world (Yin et al., 2018). The BZR family genes has been reported in sugar beet (Wang et al., 2019), *Brassica rapa* (Saha et al., 2015), Legume (Li et al., 2018), *Nicotiana benthamiana* (Chen et al., 2021), and *Pyrus bretschneideri* (Cao et al., 2020). However, the information of the evolutionary history and expression patterns of *BZRs* in wheat is limited. Consequently, in this study, a comprehensive genome-wide analysis was carried out to characterize the BZR family in wheat, and their expression profiles responses to different abiotic and biotic stresses were assessed. The present study will lay a foundation for future functional analysis of *BZR* genes in wheat, especially in response to abiotic stresses.

## Materials and methods

### Identification of wheat BZR family members

The wheat genomic data were downloaded from the International Wheat Genome Sequencing Consortium (IWGSC) RefSeq v1.1 (https://wheat-urgi.versailles.inra.fr/Seq-Repository/Assemblies/). The hidden Markov model file of the BZR protein conserved domain (PF05687.10) was downloaded from the Pfam database (http://pfam.xfam.org/search/). The known BZR protein sequences from rice (http://rice.plantbiology.msu.edu/index.shtml), *Arabidopsis thaliana* (http://www.arabidopsis.org/index.jsp), and the Markov file PF05687.10 were regarded as queries for BLASTp (e-value < 10-5) against the wheat database IWGSC.v1.1 (Han et al., 2019). The derived wheat BZRs (*TaBZRs*) sequences were then submitted to the online software Pfam (http://pfam.xfam.org/search/) to eliminate the sequences without intact BES1_N domain, which is conserved in BZR protein. The protein characteristics of *TaBZRs*, including amino acid length (aa), molecular weight (MW), theoretical isoelectric point (p*I*), instability index, aliphatic index, and hydrophilic coefficient, were analyzed by ExPASy Server10 (SIB Bioinformatics Resource Portal, https://prosite.expasy.org/PS50011).

### Phylogenetic analysis of TaBZRs

The protein sequences of TaBZRs and known BZRs from rice and *A. thaliana* were collected to construct a Neighbor-joining tree with 1000 replicated-bootstrap in MEGA7 (Kumar et al., 2016). The produced phylogenetic tree was further modified and illustrated by the online tool Tree of Life (version 3.2.317, http://itol.embl.de/) (Zhu et al., 2019).

### Gene structures and conserved motif analysis of TaBZRs

Gene structure annotations of *TaBZRs* were extracted from wheat genome annotation file. The conserved motifs of TaBZRs were analyzed by online tools MEME (v4.9.1) (Bailey et al., 2015) with the parameters setting as follows: any number of non-overlapping occurrences of each motif in each sequence; a maximum of 20 different motifs; and motif width ranging from 6 to 100 aa. The integrated image was drawn by TBtools software (Chen et al., 2020). Afterwards, the predicted motifs were analyzed by InterPro (http://www.ebi.ac.uk/interpro) (Hunter et al., 2009; Blum et al., 2021).

### Characterization of putative cis-elements in the promoter region of *TaBZR* family genes

The upstream 1500bp regions of initiation codon (ATG) of TaBZR family genes were identified by searching wheat genome data, and were analyzed to predict the cis-regulatory elements using the online program PlantCARE (http://bioinformatics.psb.ugent.be/webtools/plantcare/html/).

### Expression patterns of *TaBZRs* and real−time quantitative PCR analysis

Multiple RNA-seq data was downloaded from the NCBI SRA database, and was mapped to the wheat reference genome by Hisat2. Then, the transcripts were calculated by cufflinks. The values of fragments per kilobase of exon model per million mapped reads (FPKM) of TaBZRs were used to construct the heatmap by the R package “pheatmap”. The expression patterns of TaBZR genes under different abiotic stresses in leaf and root were detected by real-time quantitative PCR. The wheat seedlings with the first leaf fully expanded were treated with 100 mmol/L NaCl, 20% polyethylene glycol (PEG 6000), and 100 mmol/L ABA, respectively. For cold and heat treatment, wheat seedlings were put in illumination incubator setting at 4°C and 40°C, respectively. Leaf samples were collected at 0h, 6h, 12h, and 36h after treatment. The samples were immediately froze in liquid nitrogen, and then stored at -80°C for further use. Total RNA was extracted from wheat leaves and roots, and cDNA was synthesized by reverse transcriptase (Vazyme, Nanjing, China) according to the manufacturer’s instruction. Gene specific primers were designed using Primer Premier 5.0 and listed in **Table S1**. The procedure was carried out as follows: pre-denaturation at 94°C for 3 min, denaturation at 94°C for 10s, primer annealing/extension at 60°C for 30 s and collection of fluorescence signal at 72°C for 40s. The relative gene expression levels were calculated using the 2–ΔΔCt method (Livak and Schmittgen, 2001). All the experiments were performed in triplicates. Ta2291 was set as the reference gene to standardize the expression data (Paolacci et al., 2009).

## Results

### Identification of wheat *BZR* family genes

To identify the wheat *BZR* genes from the reference genome, we collected 12 known *BZR* genes from two model plants (six from rice and six from *Arabidopsis*). After removing the redundant sequences and conserved domain analysis, 20 *BZR* genes were identified in wheat (**Table 1**). Interestingly, from the results of conserved domain analysis from Pfam online tool, we found that five genes not only contained BES1_N domain, but also contained Glyco_hydro_14 domain (PF01373) in C-terminals (**Table S1**). *TaBZRs* were named according to their chromosome location. As shown in **Table 1**, *TaBZRs* were distributed on 15 chromosomes, which exhibits uneven distribution. The protein length was from 178 aa to 686 aa, of which most of the gene lengths were around 300 aa. Accordingly, the molecular weight of protein was between 19.27 KD to 75.47 KD. From **Table 1**, it is clearly that those genes having two conserved domains have the longer sequence and bigger molecular weight. The p*I* value was from 5.4 to 9.4, most of which were more than 8, suggesting that most of TaBZRs were basic proteins. Furthermore, the negative GRAVY value indicated that all of the TaBZRs were hydrophilic protein.

**Table 1:** Information of wheat BZR family genes.

Name	Accession numbers	Chr	Location	Length(aa)	MW(kD)	Domain	pI	Instability index	GRAVY
TaBZR1	TraesCS2A02G187800	Chr2A	150123521-150124807 (-)	313	33.6817	1	8.26	68.94	-0.588
TaBZR2	TraesCS2B02G219300	Chr2B	209436682-209437948 (+)	313	33.6126	1	8.26	68.28	-0.583
TaBZR3	TraesCS2D02G199900	Chr2D	151325661-151326908 (+)	313	33.6296	1	8.26	69.99	-0.586
TaBZR4	TraesCS3A02G123500	Chr3A	99587987-99588641 (+)	185	19.9684	1	9.26	50.78	-0.482
TaBZR5	TraesCS3A02G139000	Chr3A	116518912-116521151 (+)	356	37.4107	1	8.82	63.62	-0.51
TaBZR6	TraesCS3B02G142600	Chr3B	130257390-130258023 (+)	182	19.7551	1	9.18	54.86	-0.698
TaBZR7	TraesCS3B02G156600	Chr3B	149453719-149455890 (+)	354	37.2826	1	8.82	63.57	-0.517
TaBZR8	TraesCS3D02G125100	Chr3D	83703200-83703821 (+)	178	19.2675	1	9.4	56.74	-0.751
TaBZR9	TraesCS3D02G139300	Chr3D	98871878-98873985 (+)	358	37.5108	1	8.82	64.35	-0.515
TaBZR10	TraesCS4B02G009900	Chr4B	6145862-6150103 (+)	551	62.3408	2	5.4	45.63	-0.528
TaBZR11	TraesCS4D02G006100	Chr4D	3396041-3400090 (-)	686	75.4739	2	5.42	52.14	-0.37
TaBZR12	TraesCS6A02G085800	Chr6A	54115057-54120185(-)	653	73.2315	2	6.01	41.63	-0.375
TaBZR13	TraesCS6A02G338000	Chr6A	571795518-571798307 (+)	347	36.4192	1	8.79	56.05	-0.564
TaBZR14	TraesCS6B02G116400	Chr6B	101917131-101922548 (+)	672	75.4833	2	6.61	42.71	-0.348
TaBZR15	TraesCS6B02G368700	Chr6B	642915733-642918649 (+)	356	37.7176	1	8.97	57	-0.609
TaBZR16	TraesCS6D02G318800	Chr6D	427088226-427091316 (+)	348	36.4463	1	8.62	60.72	-0.516
TaBZR17	TraesCS7A02G354800	Chr7A	519131081-519133754 (+)	359	37.8497	1	8.13	63.87	-0.651
TaBZR18	TraesCS7B02G272900	Chr7B	500730357-500732768 (-)	359	37.8628	1	8.13	62.99	-0.623
TaBZR19	TraesCS7D02G368000	Chr7D	476686844-476689942 (-)	359	37.8087	1	8.13	62.55	-0.62
TaBZR20	TraesCSU02G078100	ChrUn	70282973-70290271 (+)	653	73.0873	2	6.01	41.56	-0.365

1 represents the protein only contains BES1_N domain.

2 represents the protein contains BES1_N and Glyco_hydro_14 domain.

### Phylogenetic analysis of TaBZRs

**Figure 1:**
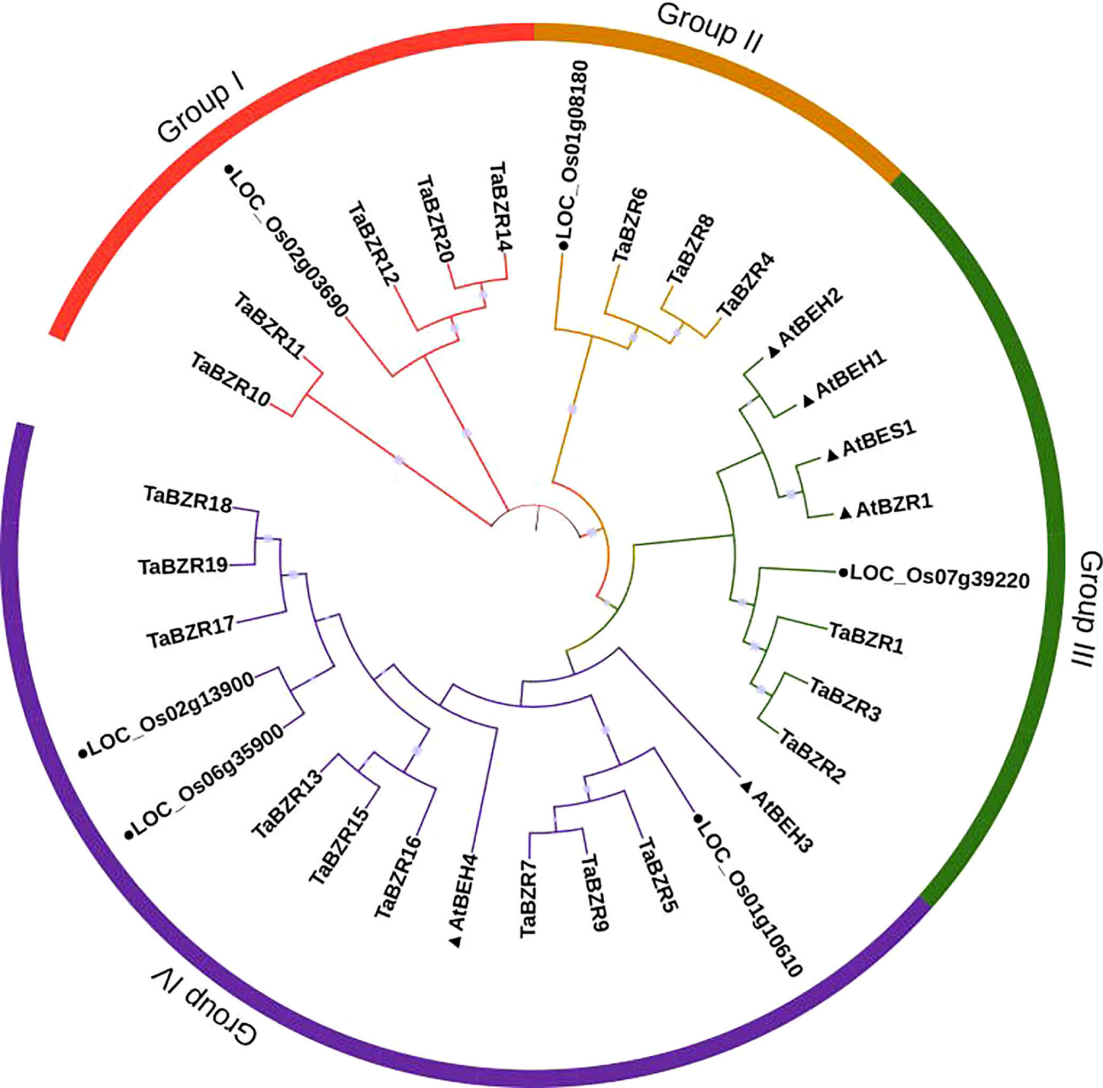
Phylogenetic analysis of protein sequences of BZRs from wheat, rice, and *Arabidopsis*. The predicted amino acid sequences were aligned by Clustalw2 sequence alignment program and a phylogenetic tree was constructed based on Neighbor-joining method with 1000 bootstrap replicates in MEGA 7 software. BZRs from rice and *Arabidopsis* were distinguished with black dot and black triangle, respectively.

To study the phylogenetic relationships of *TaBZRs*, a Neighbor-joining tree was constructed. As shown in **Figure 1**, the wheat proteins have closer relationships with proteins from rice. The total of 32 BZR proteins were divided into four groups. All the BZRs in *Arabidopsis* were clustered into group III and IV, which have more members than group I and II. All of the proteins which have two conserved domains as mentioned above (**Tables 1**, **S1**) were clustered in Group I. In addition, the triplet genes in wheat genome have the closely relationships through the phylogenetic tree.

### Motif composition and gene structure analysis of TaBZRs

**Figure 2:**
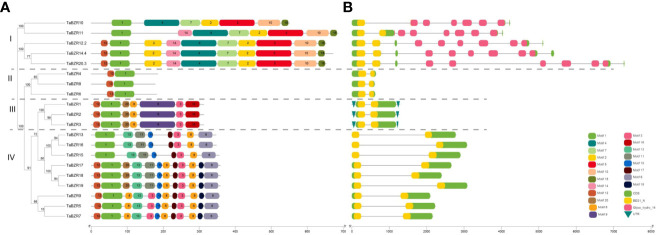
Analysis of conserved motifs, gene structure, and domains in the BZR genes of wheat. **(A)** Conserved protein domains predicted in TaBZR proteins. The conserved motifs of TaBZRs were identified by MEME tool. Each motif was represented by a colored box. **(B)** Gene structures of *TaBZR* members. Exons and introns were represented by green boxes and black lines, respectively. UTR region was represented by green inverted triangle. Conserved domains were represented by yellow (BES1_N) and pink (Glyco_hydro_14).

The distribution and composition of conservative motifs in TaBZRs were predicted by MEME online tool. As shown in **Figure 2A**, only Motif 1 presents in all TaBZRs. After analysis in Pfam (**Table S2**), Motif 1 was belonged to BES1_N domain, which is a screening criteria in present study. In addition, Motif 4, 5, and 7 were belonged to Glyco_hydro_14 domain, and only present in Group I, which were consistent with the conserved domain analysis and phylogenetic tree analysis. Furthermore, Motif 2, 10, 18 were also specifically present in Group I. In Group III and Group IV, there are also group-specific motifs: Motif 9 and 16 present in Group III; Motif 13, 15, 17, and 6 present in Group IV. Motif 3 and 8 are present in both Group III and Group IV, which indicates the close relationships of these two groups.

To study the gene structures of *TaBZRs*, we analyzed the intron-exon structure by using annotations extracted from GFF3 file and observed that all the studied *TaBZRs* were interrupted by introns (**Figure 2B**). As shown in **Figure 2B**, except for the genes in Group I, which have 7 to 10 introns, the genes in other groups only have one intron. The obvious difference between Group I and other groups is due to Glyco_hydro_14 domain presented in Group I.

### Cis-acting elements in the promoters of TaBZRs

**Figure 3:**
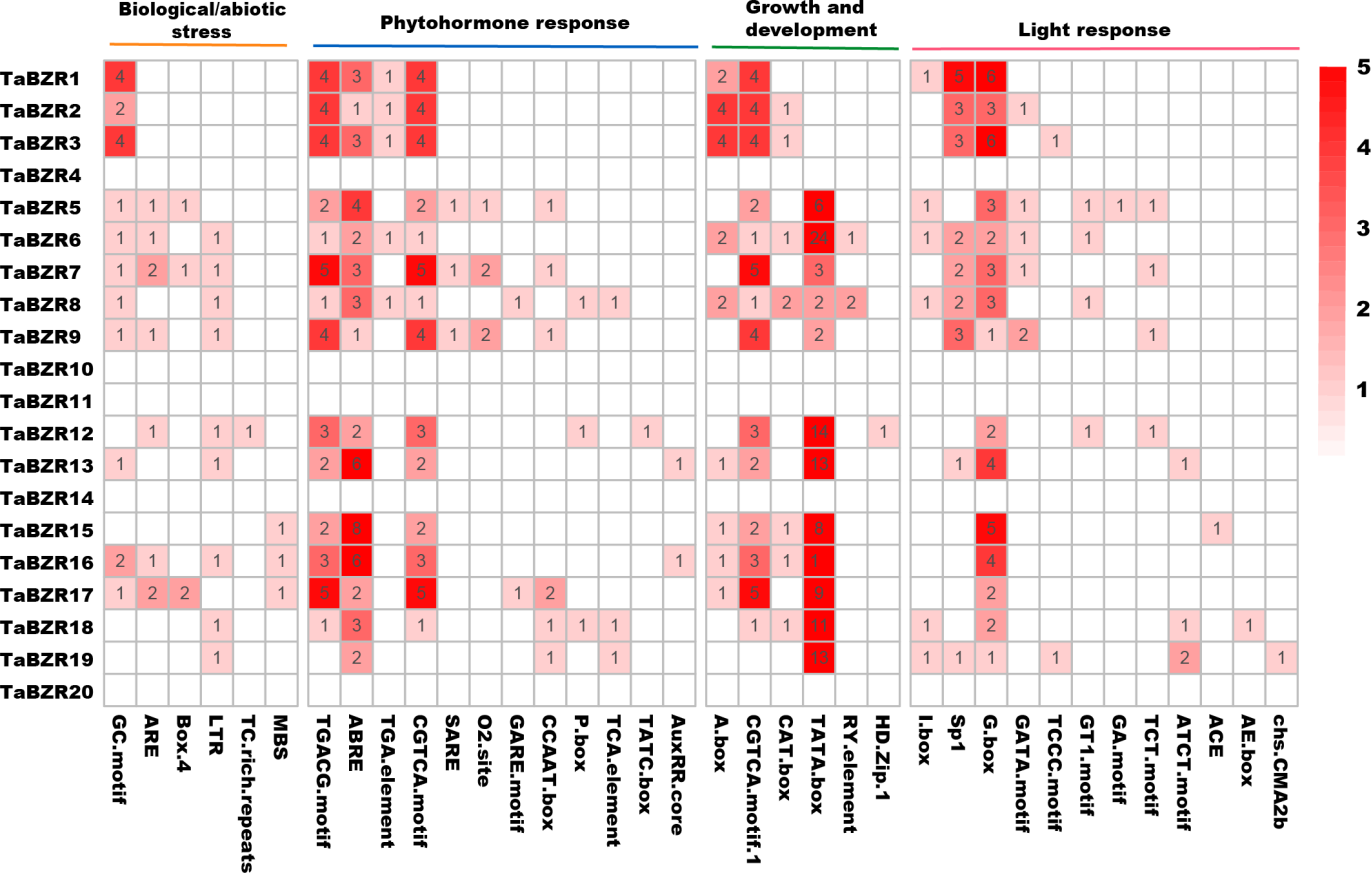
Cis-Acting elements in the promoters of *TaBZRs*. The upstream 1500bp regions of *TaBZR* family genes were analyzed to determine the cis-regulatory elements using the online program PlantCARE and the heatmap was generated by TBtools software.

In order to study the response of *TaBZR* genes to various signaling factors, we used the 1.5 kb sequence upstream of its transcription starting position to find different cis-acting elements. A total of 36 cis-acting elements were predicted (**Figure 3**; **Table S3**). It is interesting that no cis-acting elements were predicted in the promoter regions of *TaBZR4*, *TaBZR10*, *TaBZR11*, *TaBZR14*, and *TaBZR20*, which were belonged to Group I and Group II. These cis-acting elements were clustered into four categories: biotic/abiotic stress (6), growth and development (6), hormones (12), and light response (12). Results showed that the promoter sequences of *TaBZR* genes have various cis-regulatory elements. In biological/abiotic stress group, the cis-acting element GC-motif and ARE were the most common ones. The largest variety is associated with light response elements, such as GATA-motif, I-box, and G-box, among which G-box is the most abundant cis-acting element related to optical response. The cis-acting element related to abscisic acid (ABRE), auxin (AuxRR), gibberellin (GARE-motif, P-box), salicylic acid (TCA-element), and methyl jasmonate reaction (TGACG-motif), CGTCA-motif, zein metabolism (O2-site), and auxin reaction (TGA-element) were found. Six cis-elements were involved in growth and development group, in which TATA-box were the most.

### Transcriptome analysis of *TaBZRs* under abiotic stresses

**Figure 4:**
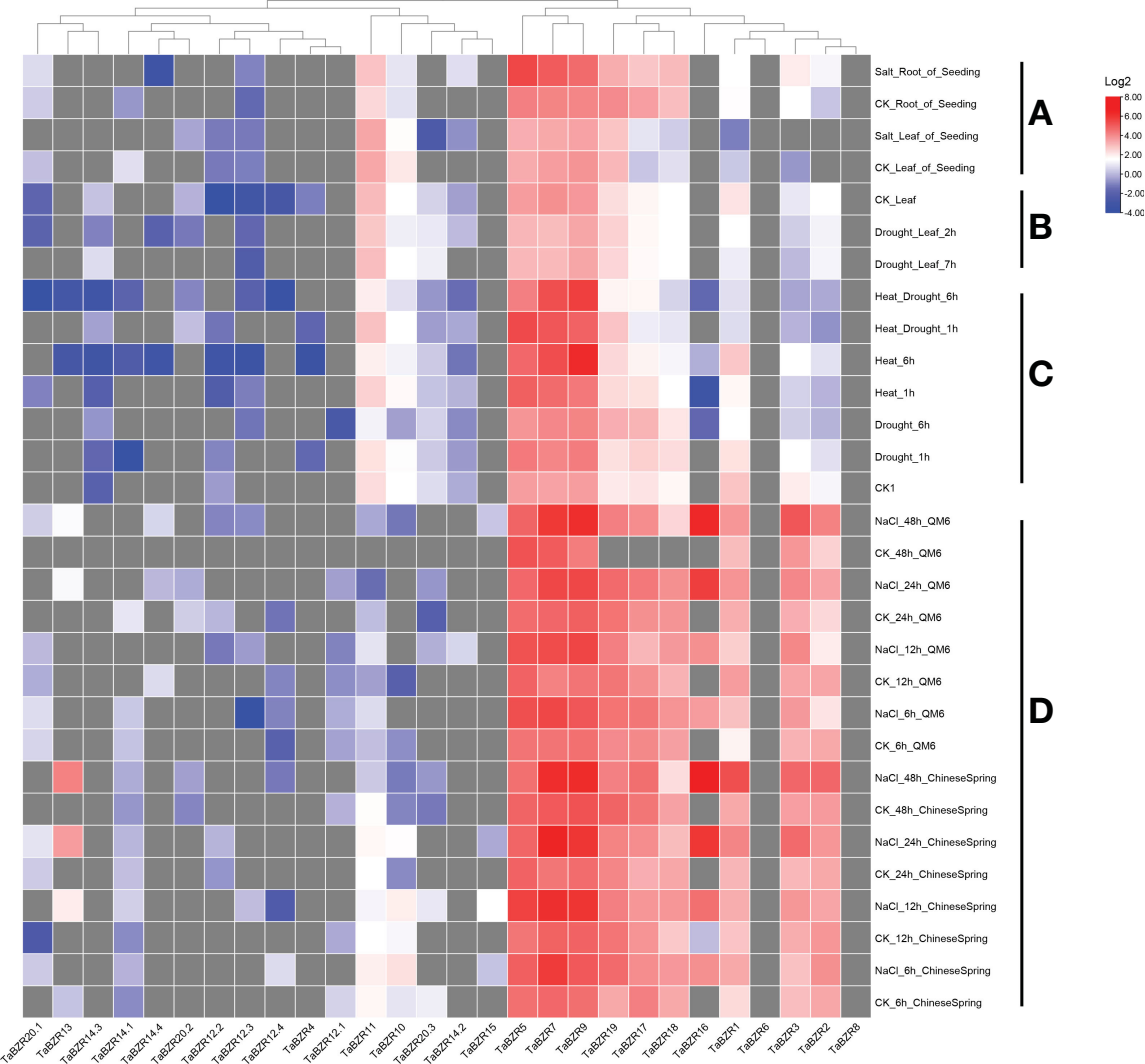
Expression patterns of *TaBZRs* under different abiotic treatments. The transcriptome raw data was downloaded from NCBI SRA database. The FPKM values were transformed by log2. The raw data of *TaBZRs* genes is provided in **Table S3**. **(A)** The expression patterns of *TaBZRs* in different tissues. **(B)** The expression patterns of *TaBZRs* after drought treatment. **(C)** The expression patterns of *TaBZRs* after drought and heat treatments. **(D)** The expression patterns of *TaBZRs* in two different cultivars after NaCl treatment.

The original RNA-seq data from multiple conditional transcriptome analysis were downloaded from NCBI SRA database and used to analyze the expression patterns of *TaBZR* genes (**Figure 4**; **Table S4**). Some *TaBZRs* has alternative splicing isoforms, so all the isoforms were included for transcriptome analysis in present study. From **Figure 4**, it was clear that the genes with different alternative splicing transcripts were almost un-expressed in the listed conditions. These results indicated that these genes might have been lost functions during evolution. On the contrary, *TaBZR5*, *TaBZR7*, and *TaBZR9* were expressed at high levels in general in all abiotic treatments. Furthermore, the expression levels of these three genes were sharply up-regulated under heat-drought treatment (**Figure 4C**) and NaCl application in QM6 and Chinese Spring cultivar (**Figure 4D**), which indicated that these three genes play important roles in wheat against abiotic stresses especially salt and heat treatment. We also found that some genes play roles in wheat resistance to salt stress, including *TaBZR1*, *2*, *3*, *17*, *18*, *16*, and *19*, especially TaBZR16 were significantly induced by NaCl application. From the above results, we can conclude that many *TaBZR* geneshave specific functions in response to salt, heat, and drought stresses.

### Expression pattern analysis of *TaBZRs* after wheat stripe rust infection

**Figure 5:**
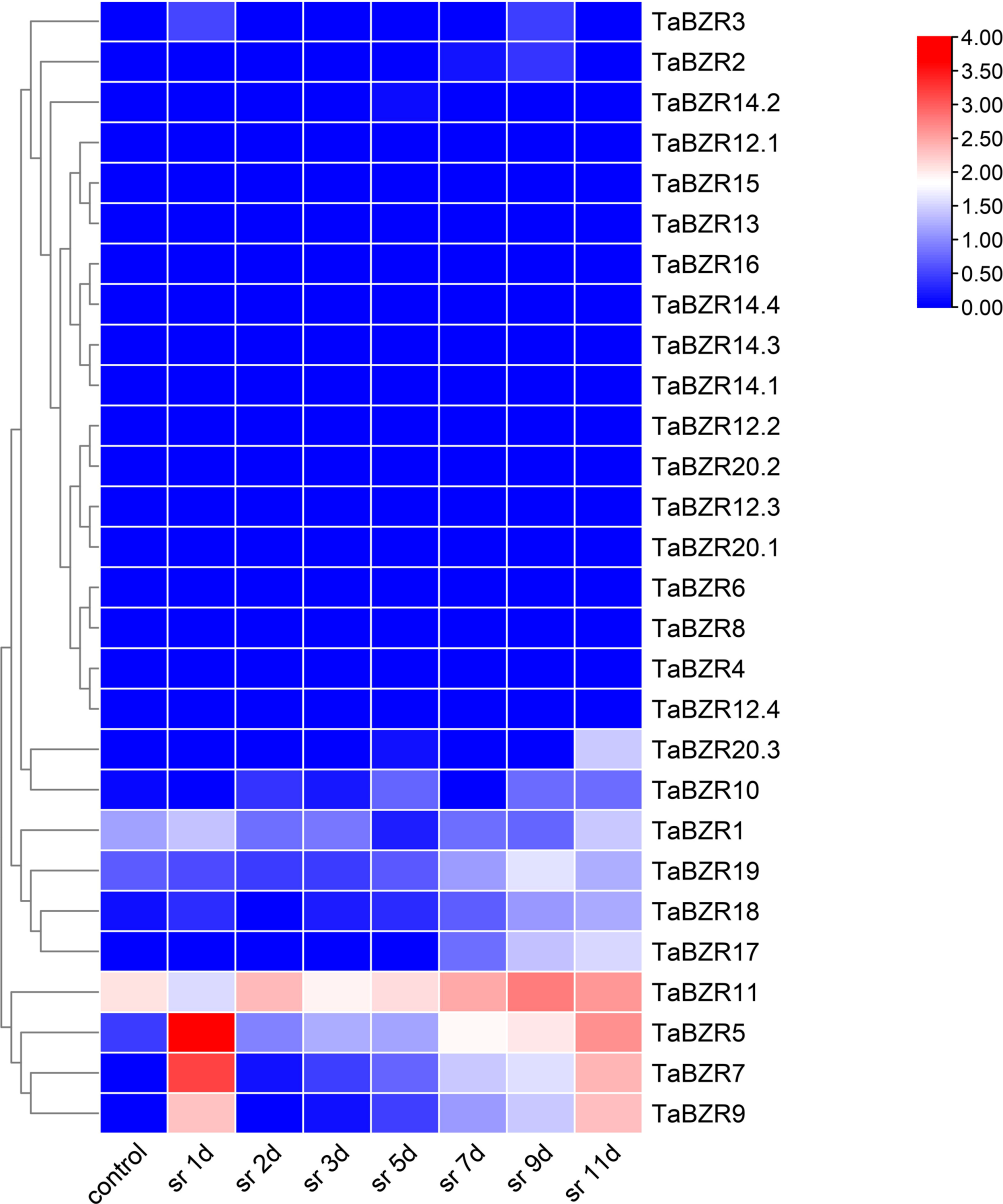
Expression patterns of *TaBZRs* after stripe rust fungus infection. The transcriptome raw data was downloaded from NCBI SRA database. The expression levels of *TaBZR* genes were provided in **Table S5**.

The expression patterns of *TaBZRs* against stripe rust infection in different time points were analyzed (**Figure 5**; **Table S5**). **Figure 5** shows the expression levels of *TaBZRs* under wheat stripe rust infection. Similar to the previous results, the genes with lower or none expression levels were still not induced by stripe rust, which solidified the above hypothesis of loss of function. The expression levels of *TaBZR5, 7, 9* were sharply rose at the first day post stripe rust inoculation, which indicated that these three genes might play important roles during the earlier stage of the interaction between wheat and stripe rust. On the contrary, the expression of *TaBZR11* was depressed at the first day, and was induced afterwards. And the expression level of *TaBZR11* reached the highest at 9^th^ day, which illustrated that *TaBZR11* might have an effect at the later stage of the wheat-stripe rust interaction.

### Real-time quantitative PCR and data analysis

**Figure 6:**
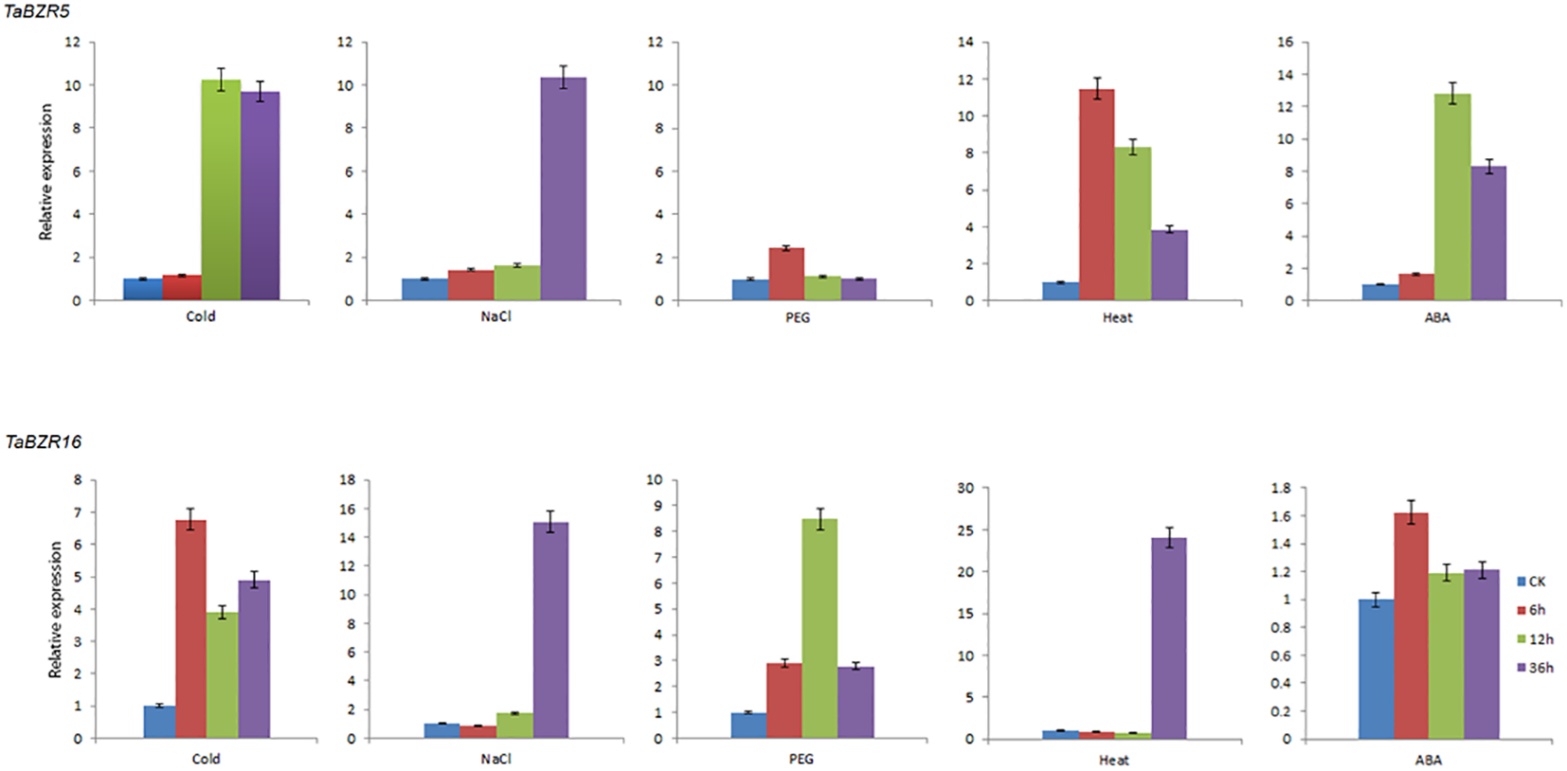
qRT-PCR analysis of expressions of TaBZR5 and TaBZR16 under five different abiotic treatments. The treatments include Cold, NaCl, PEG, Heat, and ABA spraying. Then the wheat leaves were sampled at 0h, 6h, 12h, and 36h after treatments. The expression values were calculated by 2^–ΔΔCt^ method. This experiment was carried out with three independent biological repetitions and three technical repetitions in each biological repetition.

BZR transcription factors play an essential role in stress responses (Wang et al., 2002). According to the RNA-seq data, the genes (*TaBZR5*, *TaBZR7*, *TaBZR9*, and *TaBZR16*) with high expression levels were screened out, among which *TaBZR5*, *TaBZR7*, and *TaBZR9* are triplet genes, which may have similar functions. Therefore, we further analyzed the expression patterns of *TaBZR5* and *TaBZR16* in five different abiotic stresses using qRT-PCR. The expression level of *TaBZR5* was significantly up-regulated in various conditions except for PEG treatment (**Figure 6**). In addition, the expression level of *TaBZR5* reached the peak at 6h after heat treatment, while the expression level achieved the top at 12h after cold and ABA treatment and at 36h under salt stress. However, *TaBZR16* showed different expression patterns. The expression was obviously induced after PEG treatment, while the expression levels were not obviously up-regulated after spraying ABA. These results indicated that *TaBZR5* and *TaBZR16* may play different roles and in different pathways in wheat response to cold, PEG, heat, and ABA treatments. However, similar to *TaBZR5*, the expression level reached the peak at 36h after salt treatment, which indicated that both *TaBZR5* and *TaBZR16* possibly participated in salt treatment at the same time. In conclusion, the above results indicated that members of *TaBZRs* gene family have differentiated their functions to adapt to different stresses in wheat during the process of evolution.

## Discussion

BR is a plant-specific steroid hormone that manifests a variety of stress-resistance functions and various growth-regulating effects (Lee et al., 2018). Moreover, BR signal transduction system plays an important role in plant growth and development (Kir et al., 2015). BZR transcription factors play indispensable roles in the connections between BR and other signaling pathways (Lee et al., 2018; Cui et al., 2019). It was reported that *PhBEH2* might an important hub for the crosstalk between signaling pathways in *Petunia hybrida* (Verhoef et al., 2013). However, limit genome-wide studies were performed on the BZR TF family in wheat. Identification and characterization of BZR TF family members in wheat are important starting points for exploring this gene family’s function in monocots. In this study, we used bioinformatics methods to analyze the BZR family in wheat and identified 20 *TaBZRs* (**Table 1**). Furthermore, the phylogenetic tree, gene structure, conserved motifs, and transcriptome data were analyzed. Our results will lay a foundation for further functional analysis of *TaBZRs* families.

It was reported that TaBZR2 (TaBZR9 in present study) acts as a positive regulator in drought responses by activating TaGST1 and mediates the crosstalk between BR and drought signaling pathways (Xiao-Yu et al., 2019). In present study, by analyzing transcriptome data, we found that TaBZR9 not only participated in response to drought stress, but also play important roles in wheat against salt treatment (**Figure 4**). Furthermore, recently, Bai et al. (2021) reported that TaBZR2 (TaBZR9 in present study) conferred broad-spectrum resistance to stripe rust fungus by activating a chitinase gene TaCht20.2 transcriptions. In present study, we also found that TaBZR5, 7, and 9 were up-regulated after stripe rust infection (**Figure 5**). Therefore, we can speculate that TaBZR9 might play as a hub in wheat response to various biotic and abiotic conditions, and should be given priority in further functional researches.

The expression levels of *TaBZR11* were down-regulated at 6 hours after heat and drought treatments (**Figure 4C**), which is opposite with that of *TaBZR9*. These results indicated that *TaBZR11* may play a negative role in regulating wheat against heat and drought stresses. It is interesting that TaBZR11 contains two conserved domains as mentioned before. While it is not clear whether BES1_N domain have the primary functions in these conditions. We also find out an interesting gene, *TaBZR16*, which was significantly upregulated under NaCl application (**Figure 4**), while not expressed during wheat-stripe rust fungus interaction (**Figure 5**). From this result, we can speculate that TaBZR16 specifically participated in wheat against NaCl treatment. Furthermore, it can be concluded that *BZR* genes in wheat play different roles in response to various stresses.

In this study, we performed a comprehensive genome-wide analysis of 20 wheat *BZR* gene characteristics, intron-exon structure, genomic distribution, conserved motifs, phylogeny, and expression analysis. At present, a large number of BZR genes in rice, maize, and *Arabidopsis* plants provide important information for the study of BZR genes in monocotyledonous wheat. The results of this study will lay a foundation for further in-depth functional studies of *TaBZRs* and will provide information for the breeding and genetic improvement of wheat against drought and salt stresses.

## Data availability statement

The original contributions presented in the study are included in the article/**Supplementary Material**. Further inquiries can be directed to the corresponding author.

## Author contributions

YC designed this article; PZ, HY, and YL directed the data analysis and manuscript writing. All authors contributed to the article and approved the submitted version.

## Supplementary material

Supplementary Table 3 Cis-Acting elements in the promoters of *TaBZRs*.
